# Implementation Tells Us More Beyond Pooled Estimates: Secondary Analysis of a Multicountry mHealth Trial to Reduce Blood Pressure

**DOI:** 10.2196/10226

**Published:** 2018-11-01

**Authors:** Rodrigo M Carrillo-Larco, Safia S Jiwani, Francisco Diez-Canseco, Rebecca Kanter, Andrea Beratarrechea, Vilma Irazola, Manuel Ramirez-Zea, Adolfo Rubinstein, Homero Martinez, J Jaime Miranda, GISMAL Group

**Affiliations:** 1 CRONICAS Center of Excellence in Chronic Diseases Universidad Peruana Cayetano Heredia Lima Peru; 2 Department of Epidemiology and Biostatistics School of Public Health Imperial College London London United Kingdom; 3 INCAP Research Center for the Prevention of Chronic Diseases Institute of Nutrition of Central America and Panama Guatemala Guatemala; 4 Department of Nutrition Faculty of Medicine University of Chile Santiago de Chile Chile; 5 South American Center of Excellence for Cardiovascular Health Institute for Clinical Effectiveness and Health Policy Buenos Aires Argentina; 6 Nutrition International Ottawa, ON Canada; 7 Hospital Infantil de Mexico Federico Gomez Mexico DF Mexico; 8 Department of Medicine School of Medicine Universidad Peruana Cayetano Heredia Lima Peru

**Keywords:** Argentina, behavior, clinical trial, Guatemala, health risk behaviors, lifestyle risk reduction, mHealth, Peru

## Abstract

**Background:**

The uptake of an intervention aimed at improving health-related lifestyles may be influenced by the participant’s stage of readiness to change behaviors.

**Objective:**

We conducted secondary analysis of the Grupo de Investigación en Salud Móvil en América Latina (GISMAL) trial according to levels of uptake of intervention (dose-response) to explore outcomes by country, in order to verify the consistency of the trial’s pooled results, and by each participant’s stage of readiness to change a given lifestyle at baseline. The rationale for this secondary analysis is motivated by the original design of the GISMAL study that was independently powered for the primary outcome—blood pressure—for each country.

**Methods:**

We conducted a secondary analysis of a mobile health (mHealth) multicountry trial conducted in Argentina, Guatemala, and Peru. The intervention consisted of monthly motivational phone calls by a trained nutritionist and weekly tailored text messages (short message service), over a 12-month period, aimed to enact change on 4 health-related behaviors: salt added to foods when cooking, consumption of high-fat and high-sugar foods, consumption of fruits or vegetables, and practice of physical activity. Results were stratified by country and by participants’ stage of readiness to change (precontemplation or contemplation; preparation or action; or maintenance) at baseline. Exposure (intervention uptake) was the level of intervention (<50%, 50%-74%, and ≥75%) received by the participant in terms of phone calls. Linear regressions were performed to model the outcomes of interest, presented as standardized mean values of the following: blood pressure, body weight, body mass index, waist circumference, physical activity, and the 4 health-related behaviors.

**Results:**

For each outcome of interest, considering the intervention uptake, the magnitude and direction of the intervention effect differed by country and by participants’ stage of readiness to change at baseline. Among those in the high intervention uptake category, reductions in systolic blood pressure were only achieved in Peru, whereas fruit and vegetable consumption also showed reductions among those who were at the maintenance stage at baseline in Argentina and Guatemala.

**Conclusions:**

Designing interventions oriented toward improving health-related lifestyle behaviors may benefit from recognizing baseline readiness to change and issues in implementation uptake.

**Trial Registration:**

ClinicalTrials.gov NCT01295216; http://clinicaltrials.gov/ct2/show/NCT01295216 (Archived by WebCite at http://www.webcitation.org/72tMF0B7B).

## Introduction

Mobile health (mHealth) strategies have been increasingly used in public health research studies, some of them showing effective results in improving the profiles of traditional risk factors for noncommunicable diseases [1,2], including in developing countries [3-6]. Most mHealth projects involve multifaceted complex interventions, where the interplay of their components is the key to determining an effect, thus requiring many more angles for their evaluation rather than a single indicator of effectiveness at the end of the trial [7-10].

Multisite trials are efficient in expanding enrolment targets [11,12]. Multicountry studies, in addition, can provide heterogeneity in the diversity of settings, where an intervention is being tested. It is well known that several individual and contextual factors may be directly related with the uptake and implementation of the desired intervention [13,14]. Yet, the effect of any given intervention conducted in a multisite or multicountry study may differ by site or country, and thus a single pooled effect estimate can cloud the true range of the intervention impact.

In addition to context, another major factor affecting the success of an intervention relates to the profile of participants. In biomedical and mechanistic research, the same chain of events applies to all participants. In behavioral sciences, however, it is known that targeting certain behaviors may not be the same for each person, often requiring the acknowledgment and identification of the stage of readiness to change certain habits [15,16]. The applicability of such readiness to incorporate changes toward healthier habits has been signaled for smoking cessation [17,18]. An intervention may not have the same effect on people merely thinking of quitting an unhealthy habit compared with that on those already committed to quitting.

This Grupo de Investigación en Salud Móvil en América Latina (GISMAL) trial aimed to reduce blood pressure and prevent the shift to hypertension in adult subjects with prehypertension in resource-constrained urban settings in Argentina, Guatemala, and Peru [19]. This trial used customized weekly short message service (SMS) text messages and motivational monthly phone calls aimed at the adoption of healthy lifestyles. Pooled results of the intervention showed reductions in weight but not in blood pressure after 12 months [19]. However, further scrutiny regarding the implementation of the intervention is needed to better understand what works regarding mHealth and its capability to support behavior change in real-world conditions [20]. Consequently, we aimed to conduct a secondary analysis of the GISMAL trial according to levels of uptake of the mHealth intervention to explore outcomes by country, in order to verify the consistency of the trial’s pooled results, and by each participant’s stage of readiness to change a given lifestyle at baseline. The rationale for this secondary analysis is motivated by the original design of the GISMAL study that was independently powered for the primary outcome, blood pressure, for each country.

## Methods

### Study Design

This is a secondary analysis using data collected from an mHealth randomized controlled trial (RCT) known as GISMAL (NCT01295216). The results of the trial have been reported elsewhere [19]. GISMAL was conducted in Argentina, Guatemala, and Peru by targeting adult individuals with prehypertension with the primary aim of reducing blood pressure. The intervention arm received tailored phone calls and associated SMS text messages to foster the adoption of a healthy diet and improve physical activity profiles. Phone calls using the motivational interview method were delivered monthly by nutritionists in conjunction with weekly tailored SMS text messages during a 12-month period. These communications were tailored according to the participants’ stage of readiness to change regarding the 4 hypertension-related risk factors the RCT aimed to improve, that is, physical activity, fruit and vegetable consumption, decreased consumption of high-fat and high-sugar foods, and reduced salt intake. The control arm received usual care without any mHealth components.

### Study Population

Subjects enrolled in the GISMAL trial were 30- to 60-year-old males and females with prehypertension (systolic blood pressure between 120 and 139 mm Hg, diastolic blood pressure between 80 and 89 mm Hg, or both). Further inclusion criteria were (1) those not receiving medication for hypertension; (2) those owning a mobile telephone; and (3) those able to read and understand SMS text messages in Spanish. Pregnant women and people who reported having ever been diagnosed with hypertension, diabetes, or cardiovascular disease were excluded [19].

### Variable Assessment

In total, 3 blood pressure measurements were taken in a sitting position after a 5-minute resting period using calibrated digital devices (Omron HEM-742INT, Omron Healthcare, Lake Forest, IL, United States); the mean of the last 2 readings was herein used. A digital scale (Omron SC-100/SECA 803) was used to measure bodyweight. Height was measured with the participant shoeless using a stadiometer, and waist circumference was assessed with a flexible nonelastic measuring tape. All other variables were assessed using standardized and validated questionnaires including the food frequency questionnaire [21] and the International Physical Activity Questionnaire to assess diet and physical activity, respectively [22].

#### Exposure Variable

For this study, the exposure variable was intervention uptake, defined as the intervention receiving <50%, 50%-74%, or ≥75% out of the 12 planned phone calls. The reference category was the control group of the original RCT, that is, those who did not receive the mHealth intervention. The mHealth intervention also included weekly SMS text messages related to the theme of the phone call, but it is difficult to ascertain SMS text message reception. Therefore, it was decided not to consider the SMS text messages as part of the mHealth intervention in the exposure categories. This was not considered a weakness in the ascertainment of exposure as SMS text messages were only enacted following the completion of a phone call.

#### Outcome Variables

The original trial measured the following biological and behavioral risk factors before and after the intervention: (1) systolic blood pressure (mm Hg); (2) diastolic blood pressure (mm Hg); (3) weight (kg); (4) body mass index (kg/m^2^); (5) waist circumference (cm); (6) physical activity (metabolic equivalents of task/minute per week); (7) fruit and vegetable consumption (number of daily servings); (8) high-sodium food intake (number of daily servings); and (9) consumption of high-fat and high-sugar foods (number of daily servings). For analytical purposes, these variables were treated as continuous and mean standardized (ie, the mean was subtracted from the observed values) and then divided by SD.

Regarding the analysis of these 9 outcomes, we also performed stratified analyses by country. However, when our analysis was stratified by baseline readiness to change, we chose only 4 outcomes for illustrative purposes: 2 outcomes that were expected to increase following the mHealth intervention—fruit and vegetable consumption and physical activity—and 2 outcomes that were expected to decrease following the mHealth intervention—salt added when cooking and high-fat and high-sugar food consumption.

#### Variables Used to Perform Stratified Analyses

Analyses were stratified by country (Argentina, Guatemala, and Peru) and by participants’ stage of readiness to change regarding the improvement of a particular health-related lifestyle at baseline. The 3 stages of readiness to change were constructed based on the transtheoretical model of health behavior change [23]: (1) precontemplation or contemplation; (2) preparation or action; and (3) maintenance. For the second aim of our study, based on stage of readiness to change, we used the 4 previously described health-related lifestyles, with the first 2 expected to increase and the latter 2 expected to decrease postintervention: (1) consumption of fruits and vegetables; (2) physical activity profile; (3) salt added while cooking; and (4) intake of high-fat and high-sugar foods. Of note, the transtheoretical model of health behavior change appears to be effective when implemented in culturally diverse populations [24,25].

### Ethics Approval and Consent to Participate

The protocol for the original RCT was independently reviewed and approved by Institutional Review Boards in the 3 participating countries: Hospital Italiano de Buenos Aires (Argentina), Institute of Nutrition of Central America and Panama (Guatemala), and Universidad Peruana Cayetano Heredia (Peru). The original RCT protocol was also reviewed and approved by the RAND Corporation, Santa Monica, CA, United States. Written informed consent was provided by all participants. The trial is registered at ClinicalTrials.gov (NCT01295216).

### Statistical Analysis

Numeric variables were described using means and SDs, while categorical variables were summarized using frequencies (%). Comparisons between numeric variables were assessed using analysis of variance test. Linear regression models were used, with and without stratification by country and stage of change; mHealth intervention uptake was the independent variable, and biological and behavioral risk factors (mean standardized) were the dependent variables. The regression models without any stratification were adjusted by country. The regression models stratified by country or stage of readiness did not include variables other than the exposure and outcome. Results from the regression models are presented as coefficients and 95% CIs. Analyses were conducted using STATA 13.0 (StataCorp, College Station, TX, United States).

## Results

### Principal Results

At the baseline, there were 637 participants (321 in the control group and 316 in the intervention group), 53.7% (342/637) were women, and the mean age was 43.4 (SD 8.4) years. At the end of the study, 89.4% (287/321) subjects remained in the control group and 84.2% (266/316) in the intervention group. Among those who were initially in the intervention group, 40.5% (128/316) received <50%, 36.1% (114/316) received between 50% and 74%, and 23.4% (74/316) received ≥75% of the planned intervention phone calls. There were no differences in intervention dose by sex (*P*=.05) or country (*P*=.34). However, there were more young subjects (≤45 years) among those who received either <50% or between 50% and 74% of the intervention compared with those who received ≥75% of the intervention phone calls (*P*=.001).

Multimedia Appendix 1 shows the means and SDs for each outcome of interest so that the estimates of the figures can be computed in their original units (eg, blood pressure in mm Hg). The overall unstandardized estimates are also presented in footnotes to all figures.

### Results by Country

**Figure 1:**
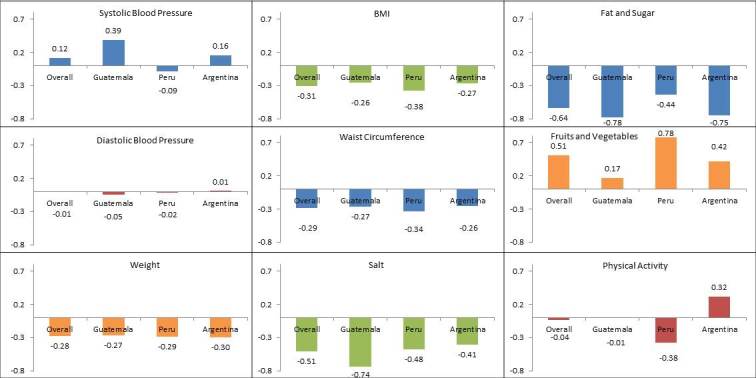
Assessed outcomes of subjects who received ≥75% of the intervention overall and by country. BMI: body mass index.

Figure 1 shows the intervention effect for each of the biological and behavioral risk factors assessed by country among subjects who received a higher dose (≥75%) of the planned intervention phone calls. Regarding all 9 metabolic risk factors, the magnitude of the intervention effect varied across countries. The intervention had an effect in the opposite direction than expected whereby systolic blood pressure increased in Argentina and Guatemala. Those in the intervention group in Peru showed a 4-fold greater fruit and vegetable consumption than those in Guatemala and almost 2-fold the consumption of those in Argentina. Moreover, the reduction of high-fat and high-sugar foods was almost 2-fold higher in Guatemala and Argentina than in Peru. Moreover, Peru appeared to be the only country where a reduction in systolic blood pressure was achieved. Overall, varying directions and magnitudes of effect were also observed among those who received <50% and 50%-74% of the intervention phone calls (Multimedia Appendices 2 and 3).

To compute each estimate in their respective units (eg, blood pressure in mm Hg), multiply SD with the reported value in the figures and then divide by the mean (Multimedia Appendix 1). The overall estimates were as follows: systolic blood pressure=123.45 mm Hg; diastolic blood pressure=74.87 mm Hg; weight=74.36 Kg; body mass index=28.85 Kg/m^2^; waist circumference=95.50 cm; salt consumption=0.40 servings/day; high-fat and high-sugar foods consumption=3.24 servings/day; fruits and vegetables consumption=2.70 servings/day; and physical activity=583.62 metabolic equivalents of task /min per week.

### Outcomes by Participants’ Stage of Readiness at Baseline

**Figure 2:**
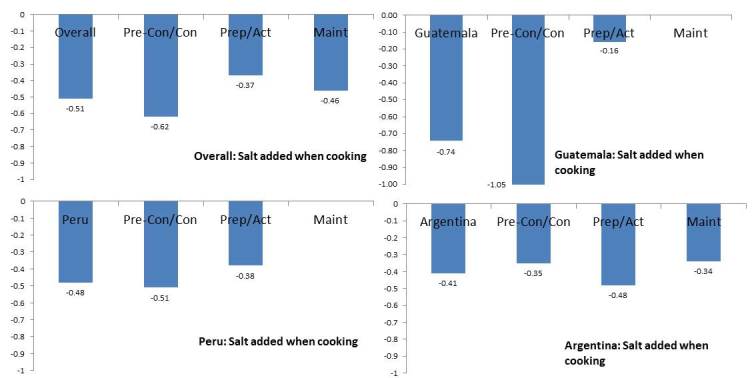
Intervention effect on salt added when cooking according to participant baseline stage of readiness status, overall and by country. Pre-Con/Con: precontemplation or contemplation; Prep/Act: preparation or action; Maint: maintenance.

After assessing the health-related lifestyle outcome of salt added when cooking (Figure 2) among those who received ≥75% of the intervention phone calls, the magnitude of the intervention effect varied according to stage of readiness to change at baseline. Specifically, the magnitude of the intervention effect was greater in those in the precontemplation or contemplation stage than in those in the maintenance stage in Guatemala and Peru.

**Figure 3:**
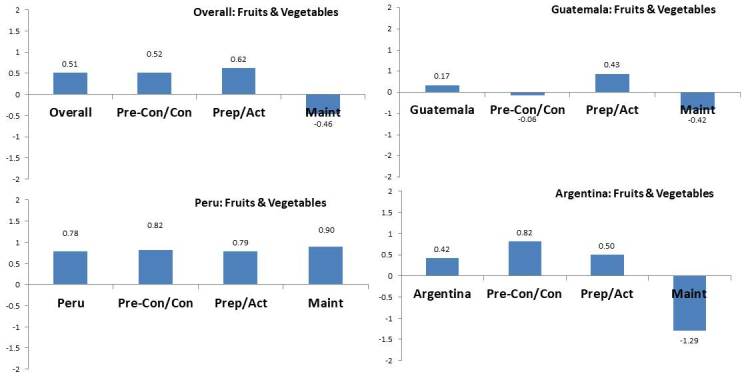
Intervention effect on fruit and vegetable consumption according to the participant baseline readiness to change status, overall and by country. Pre-Con/Con: precontemplation or contemplation; Prep/Act: preparation or action; Maint: maintenance.

Regarding fruit and vegetable consumption as a health-related lifestyle outcome (Figure 3), among those who received ≥75% of the intervention phone calls, the magnitude and direction of the intervention effect differed by country. In Peru, the direction of the intervention was the same according to stages of change, with those in the maintenance stage showing the largest effect. Overall, as well as in Guatemala and Argentina, those in the maintenance stage of readiness to change at baseline had an intervention effect that was in the opposite direction than expected, whereby fruit and vegetable consumption decreased.

The results of the intervention exposure on the other 2 health-related lifestyle factors are shown in Multimedia Appendices 4 and 5, respectively. Regarding the consumption of high-fat and high-sugar foods (Multimedia Appendix 4), Argentinians in the precontemplation or contemplation stage of readiness to change at baseline had an increased consumption that was in the opposite direction than expected. Finally, regarding the health-related lifestyle outcome of physical activity (Multimedia Appendix 5), those in the maintenance stage of readiness to change at baseline in Guatemala and Peru had decreased their physical activity profile, which was also in the opposite direction than expected.

## Discussion

### Principal Findings

Our secondary analysis of the GISMAL trial shows variations in the effect by country and stage of readiness to change at baseline. Acknowledging that the original trial was independently powered for the primary outcome—blood pressure—for each country, we found that the trial only had a positive effect on systolic blood pressure among those in Peru. Considering intervention uptake, the magnitude and direction of the intervention effect differed by country and stage of readiness to change at baseline. Among those in the higher category of intervention uptake, reductions in systolic blood pressure were also only achieved in Peru. However, fruit or vegetable intake declined among those who were at the maintenance stage at baseline in Argentina and Guatemala, respectively. These findings call for additional considerations when conducting complex multicountry or multisite behavioral interventions. For example, when planning future interventions, readiness to change could be a parameter to account for in sample size calculations among other considerations during the design stage of a study.

We reported a greater magnitude of the intervention effect among those in the precontemplation or contemplation stage of readiness to change at baseline or, in other words, those who appeared to have the least predisposition to uptake healthy lifestyles in the study sample. Those in the precontemplation or contemplation stage may have been unaware of the health risks of certain lifestyle or how to make their lifestyles healthier. However, we can speculate that the mHealth intervention provided them with the necessary amount of interaction, information, and tools to improve their lifestyles. In doing so, those in the precontemplation or contemplation stage may have been keener to engage with and uptake healthier habits compared to those in the other stages of readiness to change. Moreover, people in the precontemplation or contemplation stage had more room for improvement in terms of lifestyle behaviors; thus, even small changes in adopting healthier lifestyles were likely to have had a greater health impact than similar changes observed among those in other stages of readiness to change.

Our results from this secondary analysis align with recommendations for the need to better understand the role of technology in enacting and sustaining behavioral change [26]. The results observed in our study suggest tailoring future mHealth study interventions to specific stages of readiness to change. The duration and multipronged design of the GISMAL mHealth trial, conducted over a 12-month period targeting multiple behaviors, provides more insights into short-term action and long-term behavioral change. We did not explore predictors of engagement or habituation, which opens additional venues to understand short- and long-term effects of future mHealth behavior-oriented studies. Additional caution should be placed on the mode of mHealth delivery as it should not be assumed that all different platforms to deliver technology-based interventions would have the same adoption, engagement over time, and expected effects in the same order of magnitude [27].

From an implementation perspective, the varying results observed may have been due to differences in a number of implementation research-related indicators [20]. Among the strengths of our study lies the multicountry deployment of the same intervention allowing this mHealth intervention to operate in different the “real-world” settings. Additional strengths rely on the objective ascertainment of intervention uptake through completed phone calls made by the nutritionists, thus providing a pragmatic approach to evaluate the implementation of mHealth strategies without the need to rely on SMS text messaging (short message service, SMS) alone as a means to deliver mHealth. Fidelity was enacted before and during the conduction of the trial through a standardized approach using the same training and supervision procedure for nurse calls, the same algorithm to enact SMS text message delivery, and training of fieldworkers [19]. Appropriateness was anticipated through a qualitative study and a theory-driven development of the SMS text messages involving health communication and psychology experts before the intervention [28]. We do not have information regarding the acceptability of the intervention, which may have provided a richer picture of implementation issues. However, acceptability is in part implied by the intervention uptake over its 12-month duration, which permits a partial picture of acceptability from the end user’s point of view.

### Limitations

Some limitations include the fact that some outcomes were self-reported, such as physical activity or food consumption; specifically, salt, fruit, and vegetable consumption could have been particularly affected by self-reported information. People with higher health awareness were more likely to change their dietary behaviors but also underestimate their salt intake or overestimate their fruit and vegetable consumption. Our results could support the first hypothesis because salt intake was consistently reduced. Although fruit and vegetable consumption improved for those in different stages of readiness to change, the magnitude was relatively small and, in some cases, there was a reduction. This suggests that participants tend to underestimate unhealthy lifestyles (eg, salt consumption) rather than to overestimate the frequency of healthy lifestyles (eg, fruit and vegetable consumption). Yet, our results warrant cautious interpretation because of the lack of statistical power for subgroup analyses for all the outcomes. As the overall goal of this study was to identify signals in the effect of the intervention across countries and stage of readiness to change at baseline, we did not aim to assess such potential variations in clinically relevant units, that is, transforming standardized mean values into mm Hg for blood pressure. Lastly, a caveat of this study is the theory used to inform the methodology of the original trial [23], which has been challenged regarding its interventions to reduce smoking. Although the available evidence is inconclusive [29], some reports have suggested that the transtheoretical model could have positive results in improving weight loss and healthy diets [30].

### Conclusions

In summary, the results of this multicountry mHealth intervention trial, originally aimed at reducing the progression of prehypertension to hypertension by improving health-related lifestyles, show that outcomes vary by country and according to the participants’ stage of readiness to change a specific behavior at baseline. This information will be of utmost utility when designing future studies and provides important pragmatic lessons regarding implementation issues of mHealth interventions, emphasizing indicators amenable to be monitored.

## Appendix

Multimedia Appendix 1 Means and SDs for each outcome variable at 12 months of intervention, overall and by country.

Multimedia Appendix 2 Assessed outcomes for subjects who received <50% of the planned intervention phone calls, overall and by country. Results are. BMI: body mass index.

Multimedia Appendix 3 Assessed outcomes of subjects who received 50%-74% of the planned intervention phone calls, overall and by country. BMI: body mass index.

Multimedia Appendix 4 Intervention’s effect on high-sugar and high-fat food consumption according to the participants’ baseline readiness status, overall and by country. Precon/Con: precontemplation or contemplation; Prep/Act: preparation or action; Maint: maintenance.

Multimedia Appendix 5 Intervention’s effect on physical activity according to the participant’s baseline readiness status, overall and by country. Precon/Con: precontemplation or contemplation; Prep/Act: preparation or action; Maint: maintenance.

## Abbreviations

GISMAL: Grupo de Investigación en Salud Móvil en América Latina
mHealth: mobile health
RCT: randomized controlled trial
SMS: short message service
