# Supplementary material for: Implementation Tells Us More Beyond Pooled Estimates: Secondary Analysis of a Multicountry mHealth Trial to Reduce Blood Pressure
Source: JMIR Mhealth Uhealth. 2018 Nov 1;6(11):e10226. doi: 10.2196/10226 (PMC6238100; doi:10.2196/10226)
Supplement: Multimedia Appendix 1 [file mhealth_v6i11e10226_app1.pdf]

Supplementary Table 1: Means and standard deviations for each outcome variable at 12 month of the intervention, overall and by country.

Variable	Overall	Guatemala	Peru	Argentina	p-value
Systolic blood pressure at 12 months (mmHg)	N=533	N=170	N=193	N=190	
	122.15 (10.81)	121.18 (10.58)	119.86 (9.60)	135.34 (11.44)	<0.001
Diastolic blood pressure at 12 months (mmHg)	N=553	N=170	N=193	N=190	
	74.95 (8.23)	76.15 (7.47)	71.86 (8.05)	77.01 (8.18)	<0.001
Weight at 12 months (Kg)	N=553	N=170	N=193	N=190	
	78.62 (15.22)	74.89 (13.39)	78.74 (14.17)	81.83 (17.0)	<0.001
BMI at 12 months (Kg/m^2)	N=553	N=170	N=193	N=190	
	30.48 (5.27)	30.00 (4.96)	31.78 (4.93)	29.58 (5.64)	<0.001
Waist circumference at 12 months (cm)	N=551	N=170	N=191	N=190	
	98.94 (11.86)	96.81 (11.14)	101.59 (10.92)	98.18 (12.90)	<0.001
Physical activity at 12 months (METS/min per week)	N=536	N=171	N=184	N=181	
	632.43 (1220.30)	348.25 (531.74)	1032.55 (1736.98)	494.16 (912.13)	<0.001
Fruits and vegetables consumption at 12 months (daily servings)	N=545	N=170	N=191	N=184	
	1.97 (1.44)	1.66 (1.02)	2.45 (1.65)	1.75 (1.40)	<0.001
High sodium consumption at 12 months (daily servings)	N=550	N=170	N=191	N=189	
	0.85 (0.89)	1.15 (0.95)	0.54 (0.66)	0.90 (0.93)	<0.001
High-fat and high-sugar foods consumption at 12 months (daily servings)	N=532	N=168	N=192	N=172	
	5.01 (2.76)	5.79 (2.84)	4.18 (1.87)	5.15 (3.25)	<0.001

Results are presented as mean (standard deviation). P-value of the ANOVA test for each outcome among countries.
