# Supplementary figures and images for: Implementation Tells Us More Beyond Pooled Estimates: Secondary Analysis of a Multicountry mHealth Trial to Reduce Blood Pressure
Source: JMIR Mhealth Uhealth. 2018 Nov 1;6(11):e10226. doi: 10.2196/10226 (PMC6238100; doi:10.2196/10226)

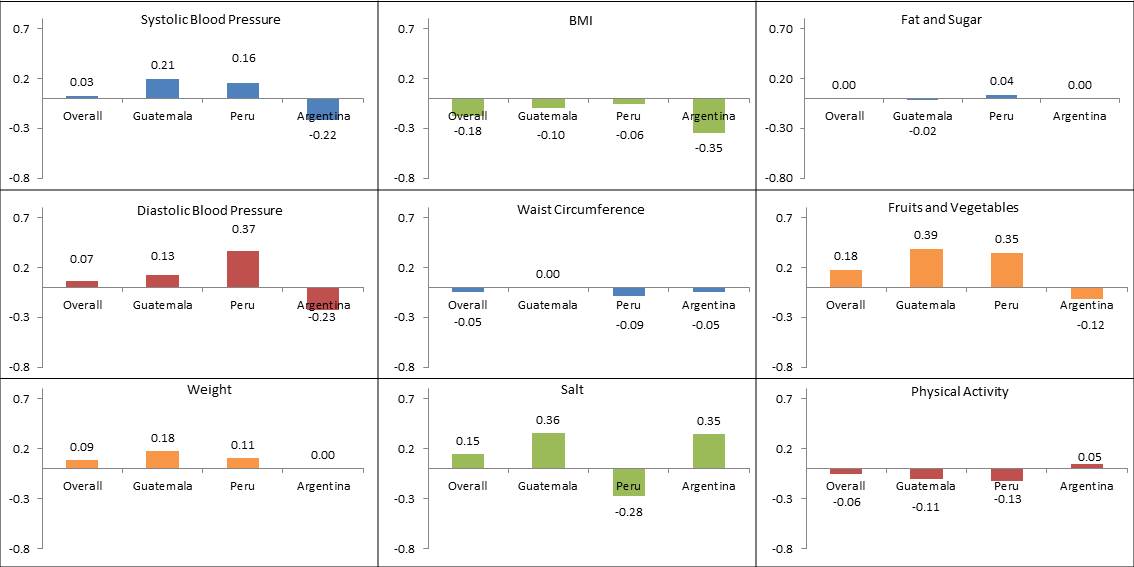

Supplement: Multimedia Appendix 2 [file mhealth_v6i11e10226_app2.jpg]

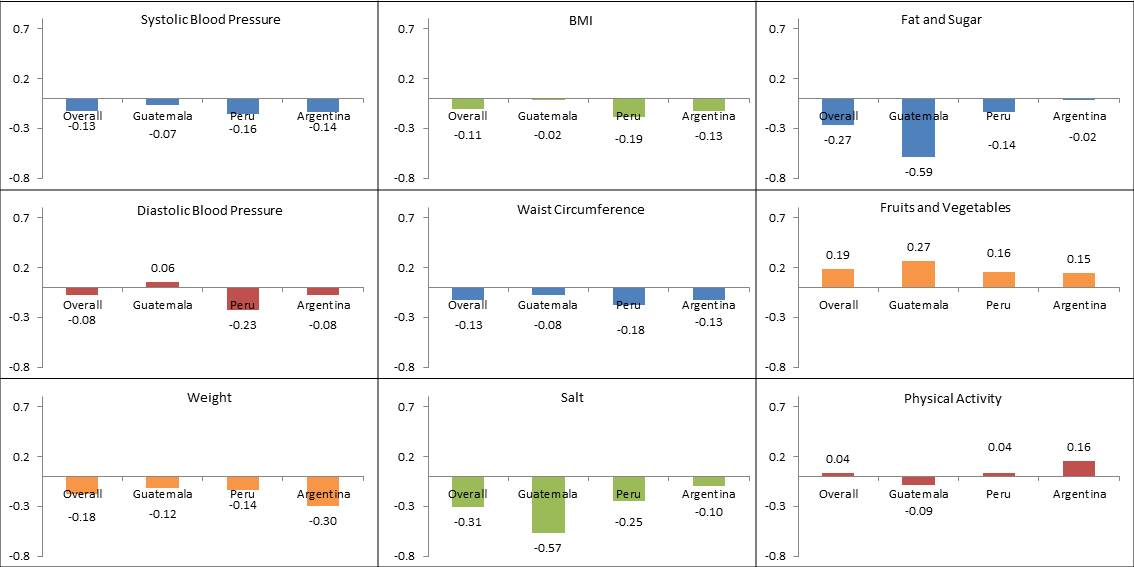

Supplement: Multimedia Appendix 3 [file mhealth_v6i11e10226_app3.jpg]

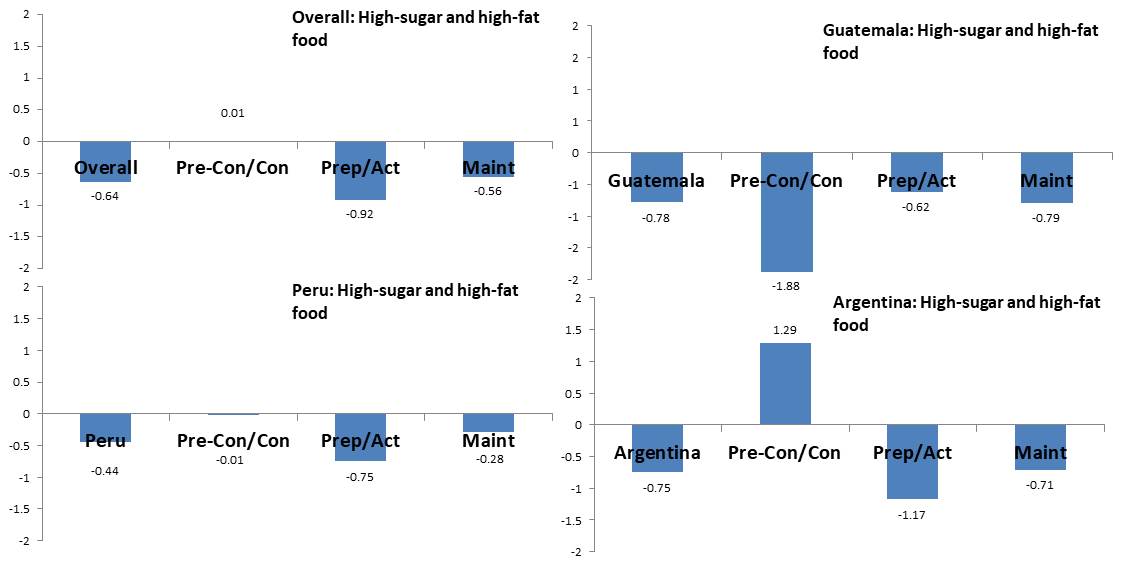

Supplement: Multimedia Appendix 4 [file mhealth_v6i11e10226_app4.jpg]

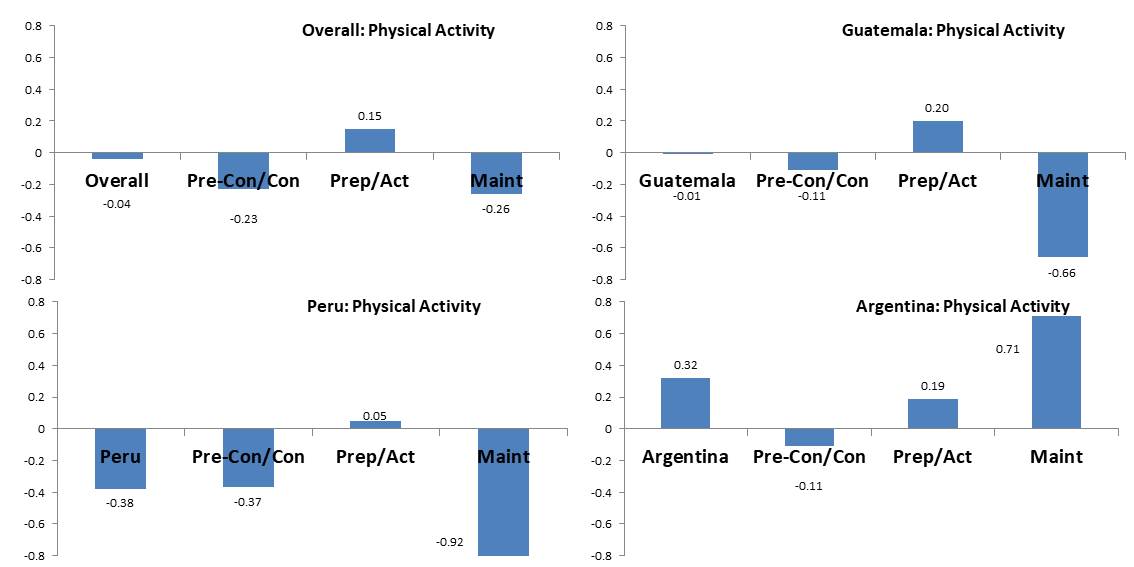

Supplement: Multimedia Appendix 5 [file mhealth_v6i11e10226_app5.jpg]
